# Optimization and experimental analysis of a cleaning device for super rice with high impurity rates based on airflow field enhancement

**DOI:** 10.1038/s41598-026-40829-4

**Published:** 2026-03-29

**Authors:** Guoqiang Wang, Fukai Wang, Yaquan Liang, Fang Li, Li Wang, Yuan Yuan

**Affiliations:** 1 Department of Agricultural Engineering, Jiangsu Agri-Animal Husbandry Vocational College, Taizhou, 225300 Jiangsu China; 2 State Key Laboratory of Intelligent Agricultural Power Equipment, Luoyang, 471039 Henan China; 3 Institute of Agricultural Facilities and Equipment, Jiangsu Academy of Agricultural Sciences, Nanjing, 210000 Jiangsu China; 4 Department of Mechanical Engineering and Mechanics, Xiangtan University, Xiangtan, 411105 Hunan China; 5 Department of Agricultural Engineering, Jiangsu University, Zhenjiang, 212013 Jiangsu China

**Keywords:** High impurity rate, Airflow field, Parameter optimization, Structural improvement, Cleaning test, Energy science and technology, Engineering

## Abstract

The harvesting process of super rice is associated with certain challenges involving the effectiveness of the harvesting equipment. To adapt the cleaning device to this process and keep pace with the performance improvements made to the separation device, the cleaning device must handle high impurity contents in the ejected material after initial separation. This paper proposes a structural improvement to the conventional wind-sieve cleaning device by incorporating streamlined arc plates with pneumatic characteristics onto the cleaning vibrating sieve, providing a dynamic airflow guidance structure to enhance the airflow distribution inside the cleaning chamber. First, indicators for evaluating the quality of the airflow field were established. Through the measurement and calculation of the airflow field, a multiobjective optimization orthogonal experiment was conducted on the working parameters affecting the airflow field. The parameter combination for the optimal performance of the cleaning device was determined to be as follows: a fan speed of 1250 r/min, first and second guide plate angles of 37° and 30°, respectively, and a fish-scale sieve opening of 24 mm. Field tests conducted to supplement the multiobjective orthogonal experiment helped confirm the reliability of the evaluation indicators for the airflow field. Based on an analysis of the airflow field, improvements were proposed for the conventional cleaning device, with aerodynamic arc plates designed to be installed on the cleaning vibrating sieve. With these structural modifications in place, more field tests were performed to verify the cleaning effect. The proposed airflow guidance structure was found to be effective in improving the cleaning efficiency, as evidenced by the reduction in the impurity rate from 4.78% to 1.78% and the loss rate from 2.46% to 0.78%. The establishment of evaluation indicators for the airflow field, structural improvements made to the wind-sieve cleaning device, and the significant reduction in the impurity and loss rates associated with rice cleaning provide practical guidance for the design of cutting and longitudinal-flow combine harvesters.

## Introduction

Rice is one of China’s most important staple crops, and its development has been significantly advanced through the Super Hybrid Rice Breeding Program over the past two decades, with super hybrid cultivars exhibiting a higher yield potential than ordinary hybrid and inbred varieties. The majority of rice fields are now harvested using combine harvesters. With the continuous increase in the rice yield and harvest efficiency, the threshing system of the combine harvester is expected to handle more amount of threshed products^1^. Field tests are also increasing in frequency and number with the significant increase in the feeding capacity of the cutting and longitudinal-flow combine harvester, placing more requirements on its threshing and cleaning performance. The threshing and separation section of the cutting and longitudinal-flow harvester adopts a front-mounted horizontal-axis flow drum, followed by one or more vertical-axis flow drums for threshing and separation. However, the cleaning device still uses the conventional wind-sieve cleaning system. Field operations have revealed that the cutting and longitudinal-flow harvester is associated with significantly high impurity and loss rates when harvesting high-yield wet rice, making it difficult to meet national standards.

The yield of rice has strong seasonal characteristics^2^, and several experiments are required to validate the effectiveness of improvements made^3^, necessitating extensive experimental verification^4^. Researchers have focused on improving the working performance of combine harvesters. For example, Yin et al.^5^ adopted a digital twin system to conduct extensive simulation tests on harvesters in a virtual environment, allowing experiments to be conducted without the limitations of operating time and harvesting scenarios. Zhu et al.^6^ improved the navigation control system of a combine harvester; Lu et al.^7^ enhanced the efficiency of field tests by modeling the working environment and optimizing the path planning of the harvester. Hao et al.^8^ revealed the vibration characteristics of the threshing drum under various abnormal operating conditions, providing a basis for the fault diagnosis of the threshing drum in combine harvesters. Park et al.^9^ constructed a dynamic simulation model of the threshing drum and evaluated the threshing performance based on the amount of rice threshed. Kang et al.^10^ designed a two-stage threshing drum with adjustable speed differences between the front and rear sections, improving the adaptability of the threshing system of the combine harvester. Liang et al.^11^ designed an optimal passive vibration isolation structure to enhance the monitoring accuracy of grain loss sensors by reducing the impact of vibrations on collision signals. Liang et al.^12^ selected a suitable fan for a rice combine harvester to achieve good cleaning performance when harvesting high-yield rice.

The two most important working processes in a combine harvester—threshing separation and cleaning—have been the main areas of research. To reduce the energy consumption during the processing and promote the design of the combine harvester, Tang et al.^13,14^ explored the structural damage patterns of rice stems during the threshing process and determined the failure thresholds for rice straw under tensile, compressive, and bending forces in both transverse and longitudinal directions. Su et al.^15^ simulated the threshing performance for rice grains under concentric and nonconcentric threshing gaps and found that the threshing effect could be significantly improved by changing the diameter of the threshing drum. Liu et al.^16,17^ pointed out that under concentric gaps, the fluctuation in the average compression force on the rice stems was lower, and compared with a concentric threshing device with a single threshing gap, a concentric threshing device with multiple gaps resulted in a lower total loss rate and improved cleaning performance. Wang et al.^18^ addressed the problem of reduced threshing performance in large combine harvesters due to the nonadjustable diameter of the threshing drum, and based on the principle of concentric adjustment, they studied a variable-diameter threshing drum with movable radial plates.

The cleaning device is a crucial component of the combine harvester, and its performance directly affects the impurity and loss rates of the grains entering the grain tank. As a conventional and classic cleaning device, the wind-sieve cleaning system has been widely studied. Jiang et al.^19^ implemented an intelligent control system for the cleaning device adopted in rice and wheat combine harvesters based on dynamic monitoring and control of the quality and operational parameters of the cleaning system. Li et al.^20^ explored the movement of materials in the airflow field and, combined with practical orthogonal experiments, established a response surface model relating operational factors to performance indicators. Liu et al.^21^ proposed an adaptive cleaning system for soybeans, which enhanced the capabilities of the wind-sieve cleaning system. Wang et al.^22^ designed a multi-wing curved centrifugal fan to evenly distribute material on the sieve surface and address clogging issues. Liang et al.^23,24^ studied the ideal airflow speed within the cleaning chamber by correlating airflow speeds at different measurement points and analyzing the cleaning performance, proposing a multi-pipeline cleaning system. During harvesting, the high moisture content of rice can influence the cleaning performance. To reduce the adhesion between wet rice threshed mixtures and improve the cleaning efficiency, Zhang et al.^25^ proposed a hot airflow cleaning method. Xu et al.^26^ introduced a hot-air drying method based on a two-stage vacuum tube dryer, which maintains efficiency while reducing the cost of the drying process.

To circumvent the high cost and cumbersome nature of conventional rice field experiments, this study measured the airflow field speed and established three evaluation indicators for the airflow quality—the average airflow speed in the pre-sieve section, the average increase in the airflow speed in the post-sieve section, and the uniformity of the lateral airflow. Multiobjective field tests and experiments were conducted to determine the optimal working parameters of the cleaning device; these helped validate the correctness of the evaluation indicators. Improvements were proposed for the vibrating sieve structure of the conventional wind-sieve cleaning device. Comparative experiments were conducted on the airflow field indicators of the cleaning device under optimal working parameters. The optimized vibrating sieve provided a dynamic airflow guidance structure for cleaning, improving the airflow distribution inside the cleaning chamber and thus enhancing the cleaning performance. Finally, field tests confirmed that the optimized vibrating sieve structure indeed reduced the loss and impurity rates associated with the cleaning device, improving its overall performance^27^. Our research results can help reduce the trial costs incurred during rice harvesting field experiments and provide practical guidance for the structural improvement of cutting and longitudinal-flow combine harvesters.

## Materials and methods

### Materials and test equipment

#### Structure and parameters of cleaning device

**Fig. 1:**
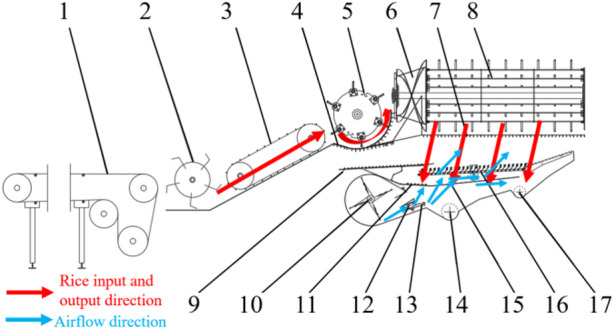
Threshing separation and cleaning testbed of a tangential-longitudinal axial combine harvester. 1. Conveyor belt, 2. Feed reel, 3. Conveyor trough, 4. Cutting flow concave plate screen, 5. Cutting flow drum, 6. Spiral feed head, 7. Longitudinal flow concave plate screen, 8. Longitudinal flow drum, 9. Upper shaking plate of the vibrating screen, 10. Fan, 11. Lower shaking plate of the vibrating screen, 12. First guide plate(air guide plate I), 13. Second guide plate (air guide plate II),14. Grain auger, 15. Braided screen, 16. Fish-scale screen, 17. Miscellaneous auger.

Pan et al.^28^ designed a work platform for a semi-fed self-propelled peanut combine harvester. Similarly, to investigate the distribution pattern of the rice threshing mixture and the airflow field characteristics inside the cleaning chamber, this study designed an experimental platform for longitudinal-flow threshing separation and cleaning by combining a ground-fixed conveyor belt and a prototype of a longitudinal-flow combine harvester^29^. The longitudinal-flow threshing separation and cleaning testbed mainly comprises the following components: a ground conveyor system, a feeding device for the conveyor trough of the cutting and longitudinal-flow combine harvester, a cutting flow threshing separation device, a vertical-axis flow threshing separation device, a cleaning vibrating sieve device, a cleaning fan system, and other accessories. Figure 1 shows the main structure of the cutting and longitudinal-flow threshing separation and cleaning testbed.

The grain threshing and separation structure comprises a cutting flow drum threshing section and a vertical-axis flow drum threshing section^30^. During harvesting, rice is conveyed through the conveyor trough into the cutting flow drum for the primary threshing. Subsequently, it is fed by the spiral feeding head into the vertical-axis flow drum for the secondary threshing. The use of the cutting and longitudinal-flow structure increases the threshing time for the rice, ensuring cleaner threshing while also achieving a higher feeding capacity. The feeding capacity of the prototype studied herein ranged from 5 to 8 kg/s.

The experimental platform was mainly used to analyze the distribution pattern of the threshed material inside the cleaning chamber of the cutting and longitudinal-flow combine harvester. During the experiment, freshly harvested rice from the field was used. Rice weighing a certain amount was taken each time and evenly spread on the conveyor belt in such a way that the rice panicles were aligned head-to-tail. The conveyor belt speed was maintained at a constant rate of 1 m/s. After initial threshing in the cutting flow drum, the rice was fed into the vertical-axis flow drum for secondary threshing under the action of the spiral feeding head at the front end of the vertical-axis flow drum^31^. During the experiment, the feeding capacity of the cutting and longitudinal-flow combine harvester was 6 kg/s, the threshing gaps of the cutting and vertical-axis flow drums were 21 and 14 mm, respectively, and the drum speeds were set to 700 and 900 r/min, respectively. Multiple repeated experiments were conducted.

#### Rice material and floating characteristics

The representative super rice variety “Zhen Dao 18” was selected for the experiments. The main component of the cleaning material was rice grain. A random sample of 100 grains was taken from the grain tank of the combine harvester to measure their geometric shape and mass^32^. The results were as follows: the average length of the rice grains was 7.28 mm, the average width was 3.36 mm, and the average thickness was 2.51 mm. The average thousand-grain weight of the rice was 24.3 g.

**Fig. 2:**
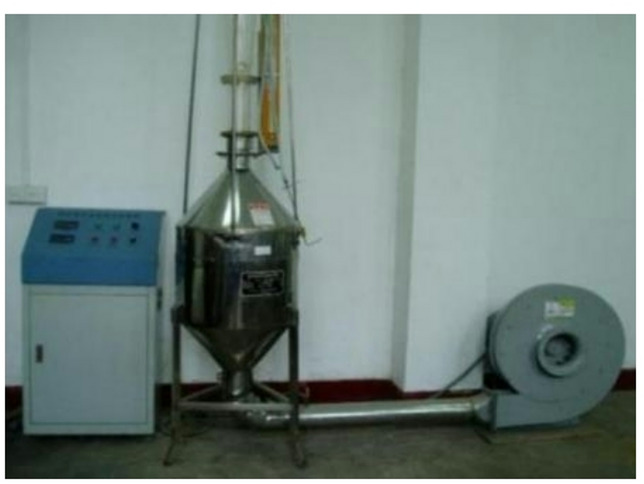
Measurement device for material floating velocity.

The material characteristics of threshed products significantly affect the stratification and sieving of the materials inside the cleaning chamber. This study aimed to optimize and improve the cleaning and sieving device from the perspective of the airflow field. Therefore, the material characteristics of each component were measured and analyzed^33^. The floating speed of each component in the cleaning material was determined using key equipment, including the DFPF-25 material floating measurement device (as shown in Fig. 2), an intelligent pressure-speed instrument, a measuring tape, and an electronic caliper. The materials to be measured were samples that entered the cleaning chamber during the operation of the cutting and longitudinal-flow combine harvester, mainly including full grains, shriveled grains, long stems (50–120 mm), short stems (20–50 mm), long grass, and light impurities (awn and broken leaves).

During the measurement, each material was measured 3 to 5 times, and the average of the maximum and minimum floating speeds was taken for each measurement. Table 1 presents the range of floating speeds for each component of the cleaning material.

**Table 1:** Floating velocity distribution of the cleaning material.

Test material	Minimum suspension speed/(m/s)	Maximum suspension speed/(m/s)
Full grains	6.18	7.78
Unfilled grains	4.32	6.62
Long stems	5.68	7.54
Short stems	4.05	7.22
Light impurities	2.51	3.12

As listed in Table 1, long stems have a suspension speed close to that of full grains, making it difficult to separate them by airflow. Moreover, the suspension speeds of the short stems and empty grains overlap with those of the full grains, which, to some extent, increases the difficulty of separation by airflow and reduces the degree of cleanliness of the grains.

#### Optimization of the guide plate structure of the cleaning Sieve

From the perspective of improving the airflow in the air ducts of the cleaning chamber of the cutting and longitudinal-flow combine harvester, this study investigated the formation of the airflow field within the cleaning cavity, which is conducive to the separation of the threshed material. Chai et al.^34^ explored the impact of guide plates on the distribution of the threshed material and cleaning losses in the cutting and longitudinal-flow combine harvester. The distribution characteristics of the threshed material in the cleaning chamber, with a significant accumulation on the upper shaking plate of the pre-sieve section, were observed. The pre-sieve section has a high cleaning load, and increasing the airflow speed in this section is beneficial for cleaning materials at the pre-sieve. Moreover, during actual harvesting operations, the vibrating sieve exhibits a reciprocating motion. Therefore, structural improvements are required to enhance the airflow guidance capability of the vibrating sieve.

**Fig. 3:**
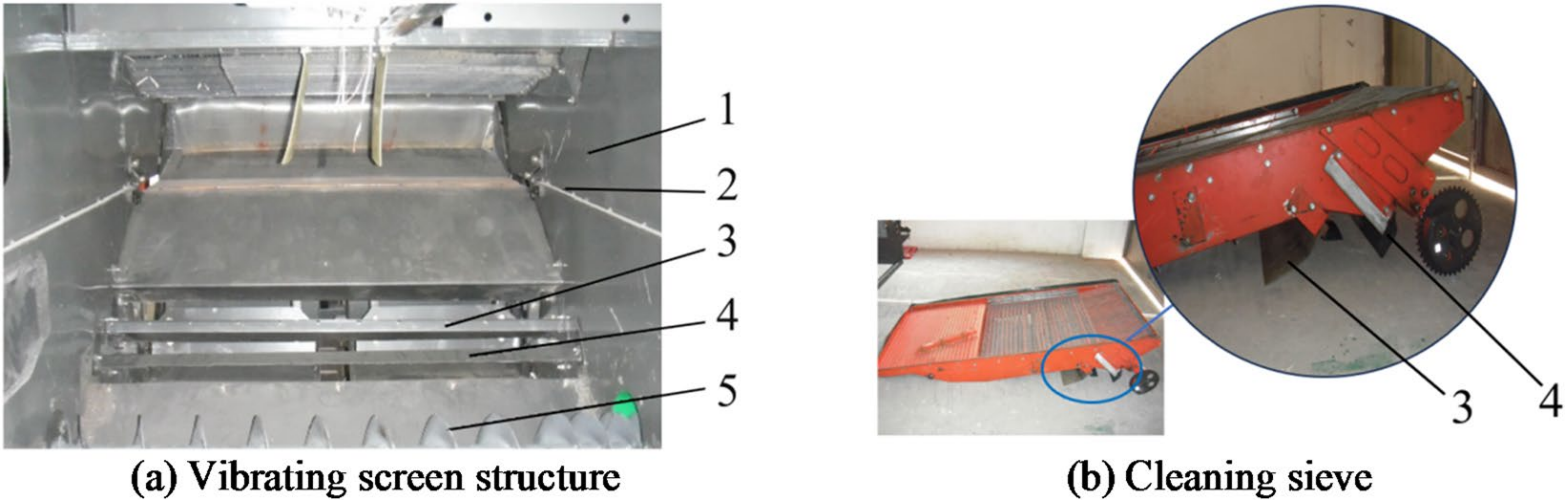
Cavity structure of the rice cleaning chamber. 1. Cleaning room walls, 2. Vibration screen installation location, 3. The first guide plate, 4. The second guide plate, 5. Grain auger.

**Fig. 4:**
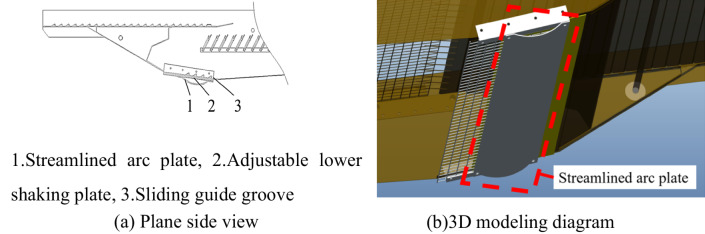
Schematics of the improved vibrating sieve structure.

As shown in Figs. 3 and 4, the structure of the vibrating screen and its installation position within the cleaning chamber reveal that the lower shaking plate plays a crucial role in airflow distribution during the cleaning process; the shape of this plate directly determines both the volume and direction of air entering the cleaning chamber.

Through an analysis of the structural characteristics of the cutting and longitudinal-flow cleaning device, to improve the cleaning efficiency of the threshed material on the upper shaking plate by increasing the airflow speed in the pre-sieve section, the structure of the lower shaking plate was optimized and improved. Figure 4 shows the improvements made to the cleaning vibrating sieve.

The lower shaking plate of the vibrating sieve adopts a combination of a length-adjustable lower shaking plate and a streamlined^35,36^ arc plate. This structure enhances the adaptability of the device for cleaning different types of threshed materials. From the analysis of the aerodynamic characteristics of the improved structure, the use of a streamlined lower shaking plate can effectively reduce the blocking effect on the airflow from the fan outlet, aiding in the formation of a high-speed upward airflow at the rear of the streamlined plate, thus increasing the airflow speed in the pre-sieve

section and improving the cleaning airflow.

### Test method for airflow field evaluation

This study employed an orthogonal experimental design to identify the optimal parameter combination, whose validity was then confirmed through field trials. Additionally, the indicators computed from the model were used to quantify the threshing performance after device improvement, and a second field experiment was conducted to verify the rationality of the modifications.

#### Test method for the distribution of rice threshed mixture

**Fig. 5:**
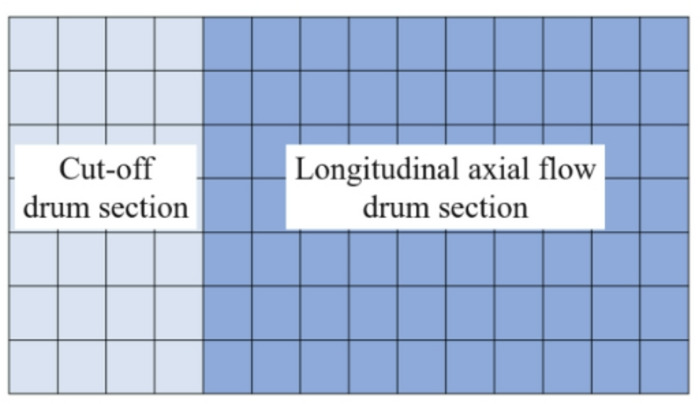
Distribution of receiving boxes for cleaning sieve material.

Grains falling into the cleaning chamber are collected in an array of receiving boxes to enable manual processing and spatial analysis of their distribution pattern. The collection area—measuring 1200 mm (length) × 980 mm (width)—is located directly above the cleaning sieve within the cutting and longitudinal-flow cleaning chamber. This area is fully covered by tightly packed receiving boxes arranged in a uniform grid: 14 boxes aligned longitudinally (along the length) and 7 boxes aligned transversely (across the width), resulting in a 14 × 7 matrix. Figure 5 illustrates the precise layout of these boxes relative to the two drum sections.

#### Arrangement of airflow field measurement points on the cleaning Sieve surface

Zhou et al.^37^ and Wang et al.^38^ proposed a grid-based sampling method to analyze the airflow distribution in the cleaning chamber, and the numerical model was validated against experimental airflow measurements. The results indicated that the measured on-site airflow velocities were in good agreement with those obtained from computational fluid dynamics (CFD) simulations, meeting the design requirements of the combine harvester cleaning system. The observed discrepancies can be attributed to the assumptions adopted in the numerical simulations, which neglected gas compressibility and viscosity and presumed a fully enclosed flow passage. In actual operation, the cleaning airflow velocity is further attenuated due to the presence of material clusters^39^.

**Fig. 6:**
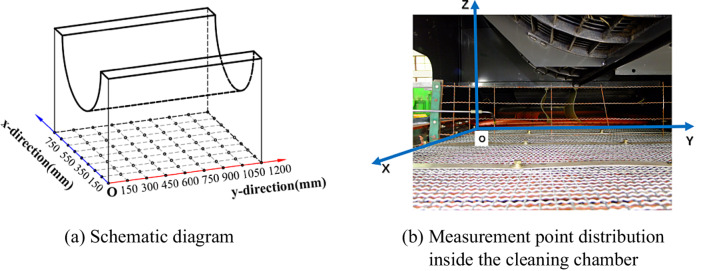
Distribution of measurement points in the cleaning chamber.

The arrangement of airflow field measurement points within the cleaning chamber was based on the intersection between the front end of the shaking plate and the left side wall of the cleaning.

chamber, which serves as the coordinate origin O. The forward direction of the machine is the negative Y-axis, and the positive X-axis is oriented vertically toward the left side wall. Figure 6(a) shows the coordinate system of the structure. Yuan et al.^40^ designed a combined cyclone separation and drum sieve cleaning system for a rapeseed combine harvester. To analyze the effect of airflow distribution on the cleaning performance of the threshed mixture entering the cleaning chamber from the threshing drum, they performed airflow field measurements on the cleaning sieve surface inside the cleaning chamber. The measurement points were arranged as follows: one plane was measured every 100 mm in the positive X-direction, with a total of nine planes; one plane was measured every 150 mm in the negative Y-direction, with a total of nine planes. The planes were parallel to the sieve surface and measured close to it. Figure 6(b) shows the coordinate system of the cleaning chamber.

**Fig. 7:**
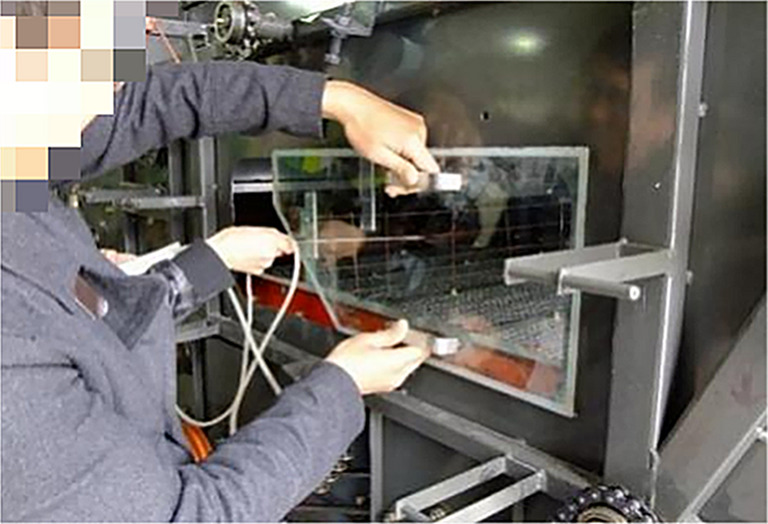
Indoor airflow field measurement inside the cleaning chamber.

The hot-wire anemometry measurements performed by Baradel et al.^41^ in a turbulent boundary layer demonstrated that turbulence spectra and velocity statistics derived from micro-wire probes exhibit good consistency. This confirms that such geometric configurations do not alter the resolved turbulent scales, implying that the effect of the velocity grid on turbulence intensity and turbulence length scales is negligible.

Air-velocity inside the cleaning chamber was measured primarily with an AVM-05/AVM-07 digital anemometer. The instrument covers 0–45 m s^–1^ with 0.1 m s^–1^ resolution and ± 3% ± 0.1-digit accuracy; it can display air volume, instantaneous value, 2/3-max flow, or peak velocity as required. To ensure that the acquired data faithfully reflect the airflow pattern, a second, intelligent pressure-type anemometer (range 0–45 m s^–1^, accuracy 0.1%) was employed during replicate tests.

Experimental procedure: Before the experiment was conducted, a safety inspection of all the working components of the prototype was performed. The measurement of the spatial points inside the cleaning chamber was conducted, and the grid points within the cleaning chamber were determined using a pre-made fine wire frame. Small short red threads were used to observe the direction of the airflow at the grid points. During the measurement, the intake of the measurement equipment was aligned with the airflow direction. To ensure that the measurements accurately reflected the airflow distribution inside the cleaning chamber, the opening of the measuring equipment installed on the side wall of the cleaning chamber was sealed with grooved transparent acrylic. Figure 7 shows the airflow measurement method inside the cleaning chamber.

#### Method for evaluating the airflow field inside the cleaning chamber

By analyzing the distribution characteristics of cutting and longitudinal-flow cleaning materials on the vibrating screen, this study defined evaluation indicators for the ideal airflow field in the combine harvester. A comprehensive assessment method based on three key indicators was established to determine whether the airflow distribution meets the optimal requirements of air-screen cleaning devices.

First, the average airflow velocity index at the front section of the cleaning sieve chamber. The analysis experiment on the material distribution pattern within the cleaning chamber revealed that the characteristic of the grains falling into the cleaning chamber from the combine harvester was that the grains expelled by the cutting flow drum tended to concentrate at the position of the upper shaking plate in the cleaning chamber. In contrast, the amount of grains expelled by the longitudinal-flow drum decreased gradually along the sieve length, while the amount of straw and other impurities increased. The airflow velocity before the sieve played a critical role in the cleaning performance. Therefore, the average airflow velocity in the front section of the sieve was defined as a key evaluation indicator.

In the coordinate system defined for the airflow field measurement experiment, the Y-direction spans the sieve area between *Y* = 400 mm and *Y* = 550 mm (corresponding to the end face of the cutting flow drum and the 200 mm region along the longitudinal-flow direction). For cleaning airflow speeds in the range of 4–7 m/s^42^, the higher the airflow velocity in the front section of the sieve, the more beneficial it is for the material to be blown away and for effective layered separation during the cleaning process.

The equation for calculating the average airflow velocity in the front section of the sieve is as follows:

1 $$\overline{w} = \frac{1}{A}\iint\limits_{D} {f_{i} (x,y)}dxdy$$

In Eq. (1), $\bar {w}$ is the average airflow velocity in the front section of the sieve, in m/s; A is the sieve surface area in the front section, in m^2^; ${f_i}$ is the fitting function of the airflow field on the sieve surface based on the spatial measurement points; D is the sieve surface domain occupied by the airflow in the front section (0 < x < 900 mm; 400 mm < y < 550 mm).

The magnitude of the airflow velocity in the front section of the sieve, as an evaluation indicator, is a significant factor when using airflow to clean the separated material. This is particularly true for cutting-flow-type combine harvesters. Since a large number of grains accumulate in front of the vibrating screen under the cutting flow drum and the impurity rate is relatively low, increasing the airflow velocity in the front section of the sieve can help quickly clean these grains. Otherwise, it would further increase the cleaning load in subsequent stages. Therefore, using the average airflow velocity in the front section of the sieve as an indicator of the ideal cleaning airflow field can serve as an important reference for designing and improving the cleaning device.

Secondly, average increase in airflow velocity at the rear section of the sieve in the cleaning chamber, as an evaluation indicator. In the cleaning chamber of the cutting and longitudinal-flow combine harvester, based on the distribution of the threshed mixture on the cleaning vibrating screen, the grain size of the material falling under the longitudinal-flow drum gradually decreases along the screen length, while the amount of large impurities, such as long and short straws, gradually increases. To efficiently and effectively separate these large impurities, a greater increase in the airflow speed at the rear section of the cleaning sieve is more favorable for expelling large impurities out of the machine, thereby reducing the impurity content in the cleaned grains. Therefore, the average increase in the airflow speed at the rear section of the sieve is defined as a key indicator for evaluating the ideal airflow field.

In the airflow field measurement experiment, within the defined coordinate system, the Y-direction covers the sieve area from *Y* = 1000 to 1200 mm. For the cutting-flow-type model, the increase in the airflow speed at the rear section of the sieve should be as large as possible.

The equation for calculating the airflow speed increase at the rear section of the sieve is as follows:

2 $$\Delta v = \frac{{\sum {v_{\max } } - \sum {v_{\min } } }}{n}$$

In Eq. (2), $\Delta \nu$ is the average airflow speed increment at the sieve tail section, in m/s; *v*_*max*_ and *v*_min_ are the maximum and minimum airflow speeds at the sieve tail section across the selected sections, in m/s, respectively; *n* is the number of selected sections.

As an evaluation indicator, the airflow speed increment at the sieve tail section is aimed at improving the separation of large foreign matter at the tail end of the vibrating screen. This indicator provides a design basis for the shape of the lower chamber of the cleaning chamber and for improving the wind guide plate structure at the fan outlet.

Thirdly, cross-sectional airflow uniformity in the cleaning chamber, as an evaluation indicator. To meet the selection requirements of the cleaning material and ensure that the material falling into the screen surface is quickly separated and passed through the sieve, Wang et al.^43^ conducted a parametric study and found that achieving good airflow distribution and uniform airflow speed in the drying chamber is crucial. From the distribution of the output material from the combine harvester on the vibrating sieve, it can be observed that the output material is evenly distributed across the same cross-section. The more uniform the airflow distribution is on the sieve at the same cross-section, the more favorable it is for the cleaning process. Therefore, the lateral airflow uniformity in the cleaning chamber is defined as an indicator for evaluating whether the airflow field distribution is ideal. The lateral airflow uniformity is calculated using the coefficient of variation^44,45^, which reflects the degree of dispersion in the instantaneous wind speed at each of the 36 points in the Y-section relative to the average speed, in order to assess the airflow uniformity in the different sections of the cleaning chamber.

The expression for the coefficient of variation (*C*_*v*_) is:

3 $$c_{v} = \frac{{\sqrt {\sum\limits_{i = 1}^{n} {\frac{{(vi - \overline{v})^{2} }}{n - 1}} } }}{{\overline{v}}}$$

In Eq. (3), *C*_*v*_ is the coefficient of variation; *v*_i_ is the actual measured wind speed at each measurement point, in m/s; $\bar{\nu }$ is the average airflow speed at the cross-section, in m/s; *n* is the number of measurement points.

Based on an analysis of the lateral airflow uniformity indicator on the sieve surface, a lower *C*_*v*_ indicates that the lateral airflow speeds are closer to the average value, resulting in a more uniform airflow distribution. Conversely, a higher *C*_*v*_ means that the lateral airflow within the chamber is more uneven, which is unfavorable for the separation and screening of the cleaning materials.

#### Field cleaning test on super rice under high-impurity-rate conditions

**Fig. 8:**
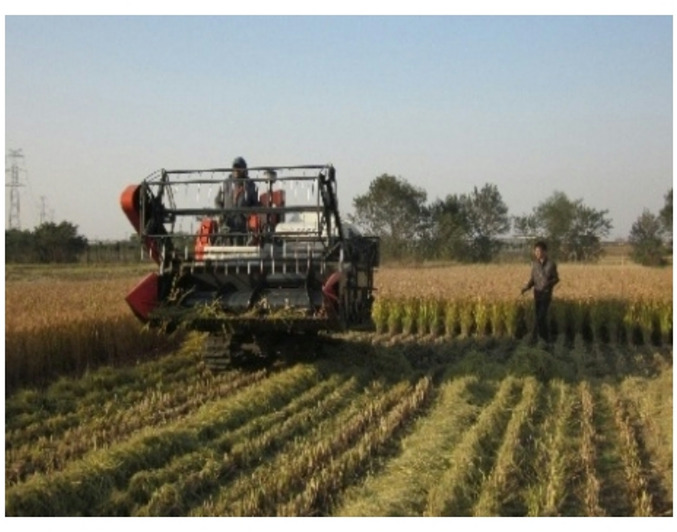
Field trial site.

The field test was conducted in mid-November 2024 at the Picheng Town rice experimental field in Zhenjiang City, Jiangsu Province. To reduce the impact of random factors on the test results, each parameter combination was repeated thrice^46^. The field validation test plan was consistent with the airflow field measurement test plan. Each set of orthogonal tests for the airflow field measurement, as well as the combinations optimized through the airflow field evaluation method, were subject to corresponding field tests, as shown in Fig. 8.

The impurity and loss rates of the grains were selected as the experimental indicators^47^.

Impurity rate: After each test, all the materials collected in the receiving boxes were weighed.

Table 2 presents the basic characteristics of the rice during the field test.

**Table 2:** Material characteristics through field measurement.

	Sampling measurement sequence number					Average value
	1	2	3	4	5	
Natural height of crop, mm	80	77	83	70	66	75.2
Head height, mm	12	14	13	14	13	13.2
Cutting height, mm	11	18	14	13	13	13.8
Grain moisture content, %	23	25	24	24.5	23	23–25
Stalk moisture content, %	67	67	68	65	64	65–67
Rice grain 1000-grain mass, g	23.8	24.2	25.1	23.9	24.6	24.32

The impurities were manually removed, and the clean grains were weighed. The percentage of removed impurities relative to the total weight of the materials was defined as the impurity content.

The grain contamination rate can be expressed as $S_{w} = \frac{{m_{1} - m_{2} }}{{m_{1} }} \times 100\%$, where ${\mathrm{~}}{{\mathrm{S}}_{\mathrm{w}}}$ is the contamination rate (%), ${{\mathrm{m}}_{\mathrm{1}}}$ is the total mass of the materials in the receiving box (g), and ${{\mathrm{m}}_{\mathrm{2}}}$ is the mass of the clean grains (g).

Loss rate: During the experiment, a mesh net was used to intercept and collect all the materials discharged from the cleaner. At the end of each trial, the grains in the discharged materials were manually collected and weighed.

The grain loss rate can be expressed as $~S_{p} = \frac{{m_{3} }}{{m_{1} + m_{3} }} \times 100\%$, where ${\mathrm{~}}{{\mathrm{S}}_{\mathrm{p}}}$ is the loss rate (%), ${{\mathrm{m}}_{\mathrm{1}}}$ is the total mass of the materials in the receiving box (g), and ${{\mathrm{m}}_{\mathrm{3}}}$ is the mass of the grains discharged from the machine (g).

The field trials were conducted using the same orthogonal experimental design as that employed for the airflow field measurement. The grain contamination rate and cleaning loss rate were used as the evaluation metrics, with the comprehensive performance (where the loss rate and contamination rate were given equal weightage) serving as the final cleaning performance indicator.

Two field experiments were conducted to validate the optimality of the parameter set and the effectiveness of the improvements.

First, validation test on airflow field multiobjective evaluation for field cleaning. The main working parameters of the cutting and vertical-flow combine harvester during field operation were as follows: The upper sieve surface uses a fish-scale sieve (with a fish-scale opening of 24 mm), and the lower sieve surface uses a woven mesh sieve (with a square hole of 25 mm), forward speed range: 1–1.2 m/s, average cutting width: 2 m.

Second, performance verification after optimization of the cleaning sieve structure for field cleaning. Owing to the improvements made to the cleaning chamber structure, airflow field measurement experiments revealed superior performance across various airflow field metrics. To ultimately verify whether the structural modifications had indeed enhanced the cleaning performance, field validation tests were designed with the contamination rate and grain loss rate after cleaning as performance indicators of the cleaning efficiency.

## Results and discussion

### Distribution of high-contamination-rate discharge from super rice

**Fig. 9:**
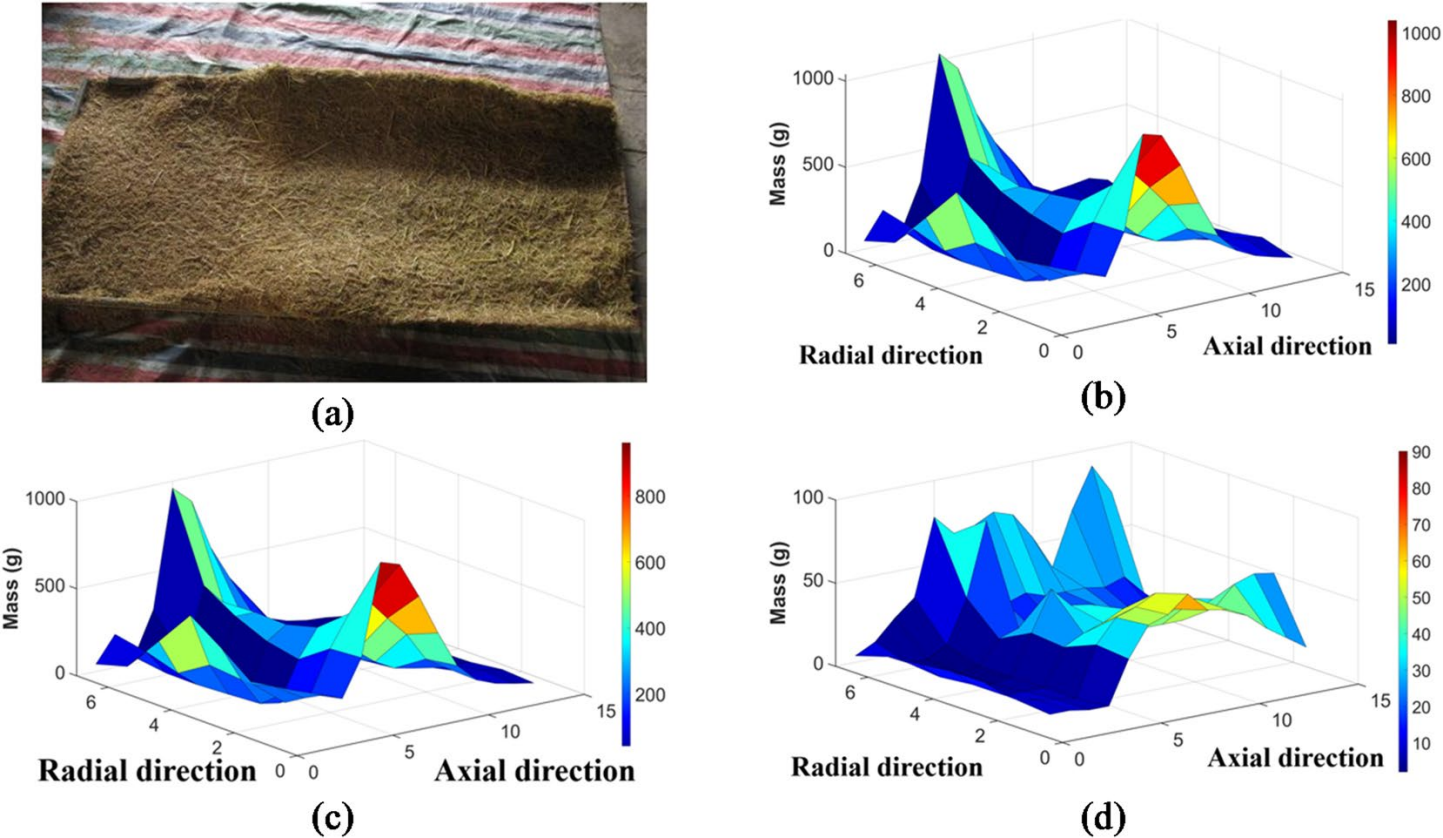
Trend distribution of the discharged materials. (a) Distribution of discharged materials. (b) Overall mass distribution. (c) Grain mass distribution. (d) Excess mass distribution.

By statistically weighing the materials collected in the 98 receiving boxes placed beneath the cutting and longitudinal-flow drum, the grain distribution along both the sieve length and sieve width directions of the vibrating screen in the cleaning chamber of the combine harvester was obtained, as shown in Fig. 9(a).

The discharged mass in each receiving box and the weight of the cleaned grains were analyzed, and a trend distribution chart was plotted using MATLAB, as shown in Fig. 9(b)–(d).

As shown in Fig. 9(b) and (c), after multiple repeated tests, the discharge distributions in each trial show a similar pattern. A large amount of grain was concentrated at the front section of the vibrating screen beneath the cutting and longitudinal-flow drum, particularly on the shaking plate, where the grains had the largest mass and were relatively clean, with minimal impurities. However, the grain mass of the discharges beneath the longitudinal-flow drum decreased along the sieve length direction, while the amount of impurities gradually increased.

The total mass of the rice discharge mixture and the mass of the rice grains along the length of the threshing drum exhibited a similar distribution pattern—a “saddle” shape. Along the axial direction of the drum, the masses of the rice discharge mixture and rice grains gradually increased, reaching a peak at the 9th receiving box (approximately *y* = 800 mm) along the axis, and then gradually decreased. This indicates that the rice discharge mixture falling onto the shaking plate from the front end of the threshing drum contained a higher proportion of rice grains, while the mixture falling from the middle and rear parts of the drum contained a comparatively lower proportion of rice grains. The distribution along the radial direction of the drum was uneven, with two peaks appearing. The mass first gradually increased, reaching a peak at the 3rd receiving box in the radial direction (approximately *y* = 400 mm), and then gradually decreased, reaching a trough at the 4th receiving box (approximately *x* = 600 mm). Thereafter, the mass gradually increased again, reaching a second peak at the 5th receiving box in the radial direction (approximately *x* = 700 mm), followed by another gradual decrease.

The distribution pattern of the impurity mass was less pronounced than those of the total mass and grain mass, as shown in Fig. 9(d). Considering the characteristics of the impurities, which are lighter and more easily blown away or dispersed by the wind, the distribution pattern was not as distinct. Along the longitudinal direction, the impurity mass gradually increased from the front to the rear of the sieve, without any significant peaks. Instead, with increasing wind speed, the impurity mass distribution shifted backward and showed a decreasing trend.

### Wind speed distribution near the sieve surface in the cleaning chamber

In conventional air-screen cleaning devices comprising a fan and a vibrating screen, the main factors influencing the airflow field quantity and direction within the cleaning chamber for this specific structure are as follows: fan speed, air intake volume at the fan inlet, fan installation angle, angles of the first and second guide plates at the fan outlet, type and opening size of the fish-scale sieve, shape of the lower cavity wall of the cleaning vibrating screen, and shape of the shaking plate beneath the vibrating screen. The fan speed and intake volume at the fan inlet mainly affected the quantity of the airflow entering the cleaning chamber from the centrifugal fan. The fan installation angle and the angles of the first and second guide plates at the fan outlet mainly influenced the direction of the airflow entering the cleaning chamber from the centrifugal fan. The type of fish-scale sieve and its opening size affected the airflow distribution in both the horizontal and vertical directions within the cleaning chamber, playing a role in guiding the airflow entering the chamber. The shape of the lower cavity wall of the cleaning vibrating screen helped guide the airflow from the second guide plate at the fan outlet, increasing the airflow speed at the rear of the sieve. The shape of the shaking plate beneath the vibrating screen: From the structure of the cutting and vertical-flow threshing cleaning test bench, it can be observed that the airflow from the centrifugal fan passes through the shaking plate beneath the vibrating screen and then enters the upper sieve cleaning chamber. This has a significant impact on the wind speed and direction on the upper sieve surface.

Wencai et al.^48^ identified that the existing seed cleaning device in vegetable seedling tray planters has a low seed cleaning performance, leading to low seedling qualification rates and high re-sowing rates. In response, they designed a seed cleaning device that integrates mechanical, air-blast, and electric control mechanisms. This paper primarily focuses on an orthogonal optimization experiment to analyze the factors significantly affecting the airflow field, such as the fan speed, angles of the first and second guide plates at the fan outlet, and opening size of the fish-scale sieve.

Table 3 presents the factor–level table, Table 4 presents the design of the orthogonal experiment table.Considering the operability of adjusting the parameters in the orthogonal experiment, four levels were set for the fan speed, the angle of the first guide plate at the fan outlet, and the opening size of the fish-scale sieve, while two levels were set for the angle of the second guide plate at the fan outlet^49^. Table 3 presents the factor–level table.

**Table 3:** Factor–level table.

Level	Factor			
	A Fan speed (*r*/min)	B Inclination angle of the first guide plate (º)	C Opening degree of the fish-scale sieve (mm)	D Inclination angle of the second guide plate (º)
1	1150	33	27.5	26
2	1050	29	24.0	30
3	1250	26	19.5	/
4	950	37	30.5	/

**Table 4:** L_8_ (2^1^ × 4^3^) Orthogonal test factors and levels.

Test sequence number	Factors and levels			
	A Fan speed (*r*/min)	B Inclination angle of the first guide plate (º)	C Opening degree of the fish-scale sieve (mm)	D Inclination angle of the second guide plate (º)
1	(1) 1150	(1) 33	(1) 27.5	(1) 26
2	(1) 1150	(2) 29	(4) 30.5	(2) 30
3	(2) 1050	(3) 26	(2) 24.0	(2) 30
4	(2) 1050	(4) 37	(3) 19.5	(1) 26
5	(3) 1250	(3) 26	(3) 19.5	(2) 30
6	(3) 1250	(4) 37	(2) 24.0	(1) 26
7	(4) 950	(1) 33	(4) 30.5	(1) 26
8	(4) 950	(2) 29	(1) 27.5	(2) 30

**Fig. 10:**
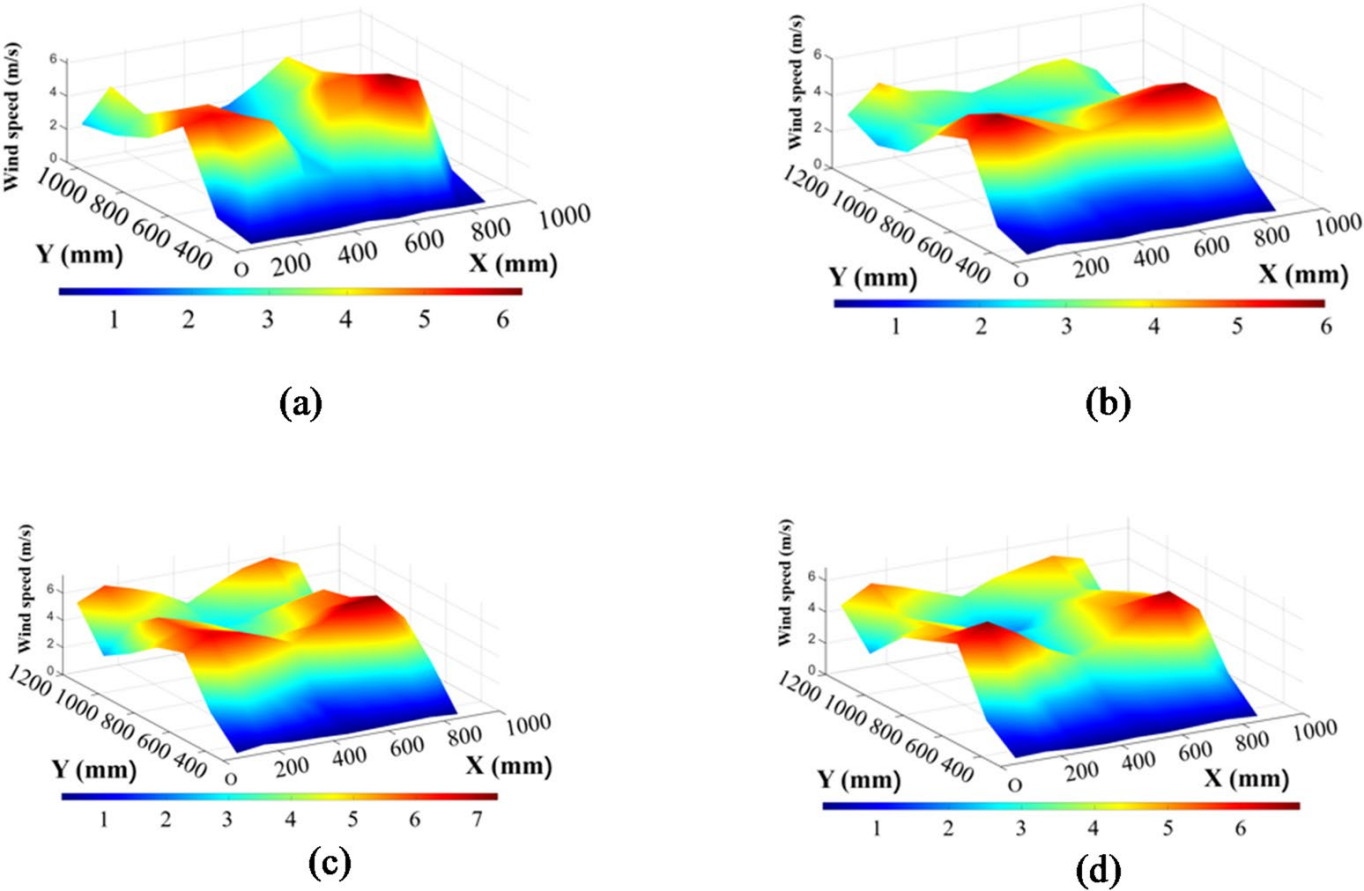
Wind speed distribution charts for Groups 1 to 4. (a) Fan speed: 1150 r/min, angle of the first guide plate: 33,opening size of the fish-scale sieve: 27.5 mm, angle of the second guide plate: 26. (b) Fan speed: 1150 r/min, angle of the first guide plate: 29,opening size of the fish-scale sieve: 30.5 mm, angle of the second guide plate: 30°. (c) Fan speed: 1050 r/min, angle of the first guide plate: 26°,opening size of the fish-scale sieve: 24.0 mm, angle of thesecond guide plate: 30°. (d) Fan speed: 1050 r/min, angle of the first guide plate: 37°,opening size of the fish-scale sieve: 19.5 mm, angle of the second guide plate: 26°.

**Fig. 11:**
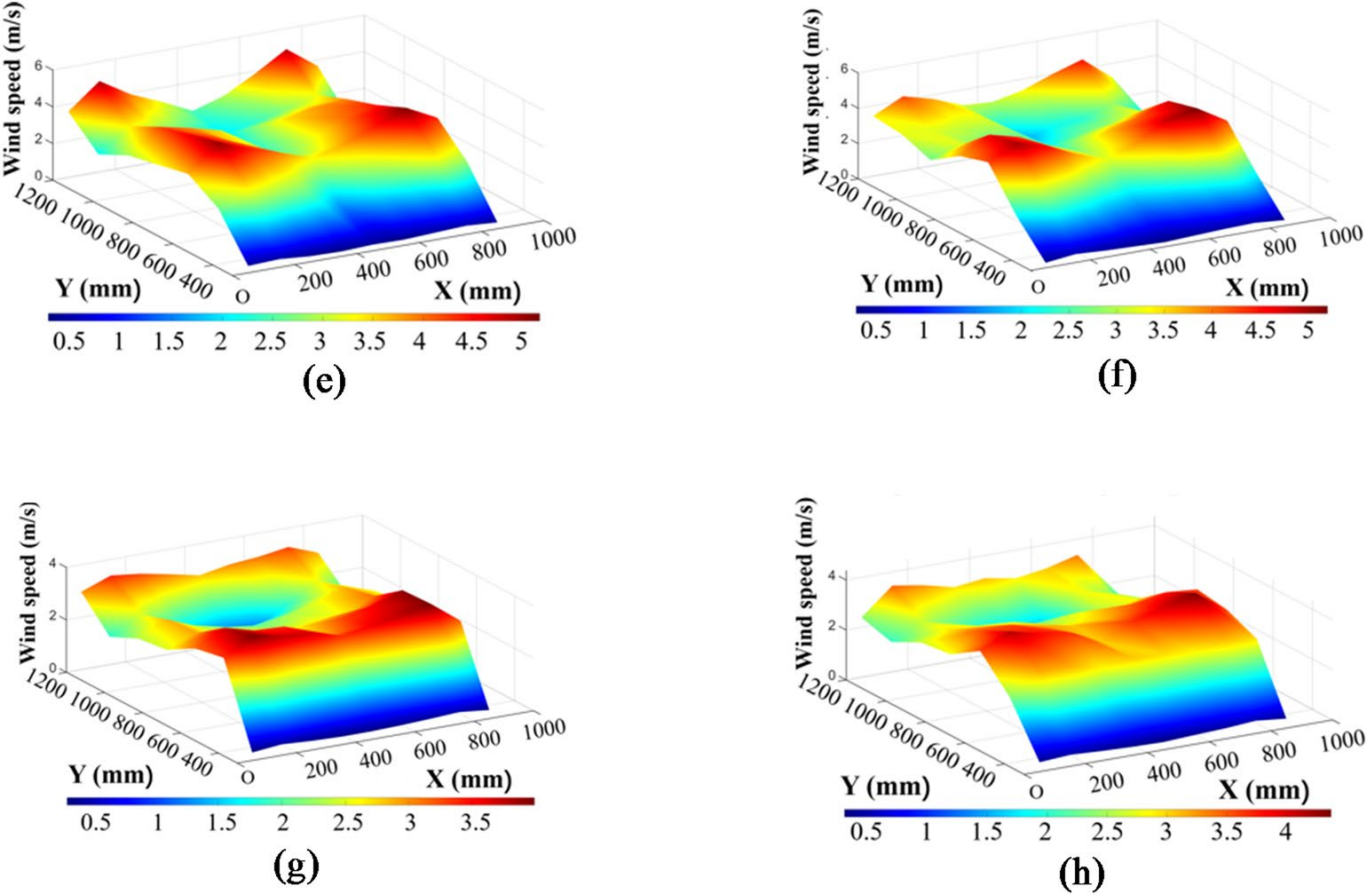
Wind speed distribution charts for Groups 5 to 8. (e) Fan speed: 1250 r/min, angle of the first guide plate: 26°,opening size of the fish-scale sieve: 19.5 mm, angle of the second guide plate: 30°. (f) Fan speed: 1250 r/min, angle of the first guide plate: 37°,opening size of the fish-scale sieve: 24.0mm,angle of the second guide plate: 26°.(g) Fan speed: 950 r/min, angle of the first guide plate: 33°,opening size of the fish-scale sieve: 30.5 mm, angle of the second guide plate: 26°. (h) Fan speed: 950r/min, angle of the first guide plate: 29°,opening size of the fish-scale sieve:27.5 mm, angle of the second guide plate: 30°.

Figs 10 and 11 show the wind speed distribution charts for the eight experimental groups.

Group 1 exhibited higher wind speeds at X = 600 mm, peaking between Y = 650–750 mm, with lower speeds at X = 300 mm and X = 1050–1200 mm. The overall wind speed distribution decreased from the center to the sides, marked by local fluctuations. In contrast, Group 2 showed greater uniformity and lower wind speed fluctuations at X = 600 mm. Moreover, Group 2 demonstrated a significantly higher wind speed in the X = 750–900 mm region, which may be attributed to the adjusted guide plate angles. Furthermore, the minimum wind speed in the X = 1050–1200 mm region was slightly higher in Group 2 than in Group 1.

Group 3 exhibited the highest wind speed of 7.31 m/s at X = 600 mm (Y = 750 mm) among all the groups. Unlike Group 4, where the high-speed zone at X = 600 mm extended toward Y = 750 mm but dropped sharply after X = 900 mm, Group 3 showed a strong wind speed gradient with a rapid decrease from X = 600 mm. The smaller fish-scale sieve opening in Group 3 likely led to airflow concentration, thereby significantly increasing the wind speed in the central region. In contrast, Group 4 had local wind speed fluctuations, and at X = 1200 mm, the wind speed increased again to a range of 4.25–5.32 m/s, probably due to the increased angle of the first guide plate. However, in the X = 300 mm region, the wind speed distribution in Group 4 was relatively stable with no extreme fluctuations.

The increased fan speed resulted in overall higher wind speeds in Group 5 than in Group 4, both of which shared the same sieve opening size. Specifically, the wind speed at X = 600 mm reached 5.16 m/s. The wind speed distribution in Group 5 was also relatively uniform, with minor differences across the X = 450–900 mm region and no significant peak values. Moreover, a notable decrease in the wind speed was observed near the cleaning chamber wall at X = 1200 mm compared with Group 4.

Group 6 showed a medium-to-high wind speed zone concentrated in the X = 600–750 mm range without extreme peaks. Group 7 had lower overall wind speeds with minimal differences across X = 450–900 mm and no extreme peaks. Compared with Group 7, Group 8 showed higher wind speeds at X = 600 mm under the same fan speed, along with a more uniform distribution in the X = 450–750 mm range.

### Multiobjective orthogonal experiment and field cleaning performance

#### Multiobjective orthogonal experiment on airflow field

Given the variety of airflow indicators, three airflow-field indices are designed according to the characteristics of the data. The mean airflow velocity at the front section of the screen was calculated as follows. Based on the measured velocity values inside the cleaning chamber, the air-speed distribution over the plane adjacent to the sieve surface was fitted with a polynomial function:

4 $$y={a_0}+{a_1}x+{a_2}{x^2}+ \cdots +{a_n}{x^n}$$

using the MATLAB curve-fitting toolbox^50^. The resulting fitted surface was then integrated over the region 400–500 mm in front of the screen to obtain the average velocity; the values are listed in Table 5.

Subsequently, the increase in the airflow velocity in the post-screening section was calculated based on the definition described in the previous section. Table 6 presents the airflow speeds in the front section of the sieve and the average increase in the airflow speed in the rear section for each group of orthogonal experiments.

**Table 5:** Summary of polynomial fitting results for the average airflow velocity.

Test No.	Polynomial fit of the mean airflow velocity	*R*-squared	Pre-screening airflow velocity$\bar{w}$/ m/s
1	${\mathrm{v}} \approx {\mathrm{-14}}{\mathrm{.79+0}}{\mathrm{.06x+0}}{\mathrm{.07y}}$	0.70028	3.59
2	${\mathrm{v}} \approx {\mathrm{-10}}{\mathrm{.26+0}}{\mathrm{.047y}}$	0.77225	3.31
3	${\mathrm{v}} \approx {\mathrm{-11}}{\mathrm{.52-0}}{\mathrm{.03x+0}}{\mathrm{.06y}}$	0.81411	3.32
4	${\mathrm{v=-11}}{\mathrm{.29+0}}{\mathrm{.05y}}$	0.77936	3.48
5	${\mathrm{v}} \approx {\mathrm{-10}}{\mathrm{.04+0}}{\mathrm{.05y}}$	0.84322	4.43
6	${\mathrm{v}} \approx {\mathrm{-11}}{\mathrm{.47+0}}{\mathrm{.06y}}$	0.83182	4.68
7	${\mathrm{v}} \approx {\mathrm{-15}}{\mathrm{.72+0}}{\mathrm{.08y}}$	0.8627	3.05
8	${\mathrm{v}} \approx {\mathrm{-12}}{\mathrm{.14+0}}{\mathrm{.06y}}$	0.93438	2.95

**Table 6:** Average increase in the airflow velocity at the rear section of the sieve.

Test No.	1	2	3	4	5	6	7	8
$\Delta \nu$ /m/s	0.83	1.07	1.17	1.16	1.51	1.54	0.91	1.16

Finally, the uniformity of the transverse airflow in the longitudinal shear flow was analyzed based on the measured data. The coefficient of variation (*C*_*v*_) for the airflow at different longitudinal sections of the sieve (*Y* = 450, 600, 750, 900, and 1050 mm) is calculated. To facilitate the further analysis of the orthogonal experiment, the average *C*_*v*_ value for the five selected sections was taken^51^, represented as *C*_v_*. Finally, the lateral airflow uniformity in the entire cleaning chamber is evaluated using the average C_v_* for each group. Table 7 presents the lateral airflow average coefficient of variation *C*_v_* under the orthogonal experiment.

**Table 7:** Cross-flow variation coefficient and mean value table under different parameters.

Test No.	C_v_ of the cross-section along the y direction /mm					
	450 C_450_	600 C_600_	750 C_750_	900 C_900_	1050 C_1050_	Average Cv^*^
1	0.611	0.531	0.752	0.645	0.521	0.612
2	0.512	0.471	0.742	0.423	0.458	0.482
3	0.357	0.321	0.523	0.243	0.231	0.325
4	0.379	0.376	0.567	0.423	0.361	0.421
5	0.471	0.592	0.762	0.485	0.346	0.531
6	0.336	0.325	0.548	0.312	0.202	0.345
7	0.335	0.471	0.397	0.435	0.239	0.387
8	0.245	0.498	0.587	0.346	0.329	0.361

Orthogonal Experiment Optimization Analysis of the Four Working Parameters Based on Ideal Airflow Field Indicators. By calculating the defined indicators for measuring the ideal airflow field, an orthogonal experiment optimization analysis was conducted on the four working parameters under consideration. Considering the operability of adjusting the four factors during the experiment, the levels of the four factors were set differently. The range values were converted according to Table 8 and then optimized using the range method^52^.

**Table 8:** Conversion coefficients for various orthogonal test levels.

Number of levels	m	2	3	4	5	6
Conversion coefficients	*d*	0.71	0.52	0.45	0.4	0.37

The range conversion relationship is $R^{\prime} = dR\sqrt r$, where *r* is the repetition count for each factor level, *d* is the conversion coefficient, which is related to the factor levels, and *R*’ is the range after adjustment.

Based on the three aforementioned indicators, a multi-objective orthogonal experimental array is designed to evaluate the improvement effect. Based on the aforementioned orthogonal experimental analysis method, optimization experiments were conducted using indicators such as the lateral airflow uniformity, average airflow velocity at the front of the screen, and airflow recovery at the tail of the screen. The objective was to find the optimal combination of the four structural parameters: fan speed (A), first guide plate angle (B), fish-scale sieve aperture (C), and second guide plate angle (D). Table 9 presents the orthogonal experiment results of the airflow field.

Based on an analysis of the lateral airflow uniformity presented in Table 8, the main factor affecting the airflow uniformity in the cleaning chamber of the longitudinal-flow combine harvester was found to be the angle of the first guide plate (B1), followed by the aperture of the fish-scale sieve (C). The optimal combination for optimizing the lateral airflow uniformity was A2C2B4D2.

Regarding the analysis of the average airflow velocity at the front of the screen, the fan speed (A) had the greatest influence, followed by the angle of the first guide plate at the fan outlet (B1). The optimal combination of working parameters for achieving the best average airflow velocity at the front of the screen was A3B4D1C2.

Based on the optimization using the average increase in the airflow velocity at the rear of the screen as the evaluation criterion, it can be concluded from the table analysis that the fan speed and the angle of the second guide plate at the fan outlet were the most significant factors. The optimal parameter combination was A3D2B4C2.

Due to the significant differences in the impact of the four factors on the three indicators, a comprehensive balancing method was applied to analyze the orthogonal experiment results^53^. The factors were ranked based on the importance of each indicator, with the most influential factor ranked first, followed by the second most influential, and so on. Table 10 presents the factor importance order and optimal levels.

**Table 9:** Orthogonal experiment results.

	j	*i*				Pre-sieve airflow Yi	Rear sieve airflow Ki	Cross-sectional airflow uniformity Ti
		A Fan speed	B Inclination angle of the first guide plate	C Opening degree of the fish-scale sieve	D Inclination angle of the second guide plate			
	1	1	1	1	1	3.59	0.83	0.612
	2	1	2	4	2	3.31	1.07	0.482
	3	2	3	2	2	3.32	1.17	0.325
	4	2	4	3	1	3.48	1.16	0.421
	5	3	3	3	2	4.43	1.51	0.531
	6	3	4	2	1	4.68	1.54	0.345
	7	4	1	4	1	3.05	0.91	0.387
	8	4	2	1	2	2.95	1.16	0.361
Cross-sectional airflow uniformity indicator	Tj1	1.10	1.00	0.97	1.77			
	Tj2	0.75	0.84	0.67	1.70			
	Tj3	0.88	0.86	0.95				
	Tj4	0.75	0.77	0.87				
	Rj	0.35	0.23	0.30	0.07			
	R’	0.22	0.15	0.19	0.09			
	Optimal parameter combination	ACBD A2C2B4D2						
Pre-sieve airflow indicator	Yj1	6.9	6.64	6.54	14.8			
	Yj2	6.8	6.26	8	14.01			
	Yj3	9.11	7.75	7.91				
	Yj4	6	8.16	6.36				
	Rj	3.11	1.9	1.64	0.79			
	R’	1.98	1.21	1.06	1.12			
	Optimal parameter combination	ABDC A3B4D1C2						
Rear sieve airflow indicator	Kj1	1.9	1.74	1.99	4.44			
	Kj2	2.33	2.23	2.71	4.91			
	Kj3	3.05	2.68	2.67				
	Kj4	2.07	2.7	1.98				
	Rj	1.15	0.96	0.73	0.47			
	R’	0.73	0.61	0.47	0.67			
	Optimal parameter combination	ADBC A3D2B4C2						

**Table 10:** Factors of the primary and secondary order and corresponding optimal levels.

Indicators	Primary and secondary order	Optimal level
Cross-sectional airflow uniformity indicator	A C B D	A2C2B4D2
Pre-sieve airflow indicator	A B D C	A3B4D1C2
Rear sieve airflow indicator	A D B C	A3D2B4C2

As shown in Table 10, Factor A has the greatest impact on the three indicators, followed by factor B, and lastly Factor C. The impact of each level of the four factors on the indicators is also different. By counting how many times each level of the factors is selected as the optimal level for each indicator, the optimal levels of the factors could be determined. The final result shows that the optimal airflow field combination for rice cleaning was A3B4D2C2.

#### Cleaning performance test under different working parameters

The analysis results of the airflow field were compared and verified through a field orthogonal test. Table 11 presents the test results.

**Table 11:** Results of the field orthogonal test.

j	i				y* performance /%	yi1 Impurity rate /%	yi2 Loss rete%
	A Fan speed/ (*r*/min)	B Inclination angle of the first guide plate / (°)	C Opening degree of the fish-scale sieve / (mm)	D Inclination angle of the second guide plate / (°)			
1	1	1	1	1	3.77	4.01	3.55
2	1	2	4	2	2.35	2.65	2.11
3	2	3	2	2	3.40	3.59	3.23
4	2	4	3	1	3.55	3.74	3.38
5	3	3	3	2	3.12	3.89	2.61
6	3	4	2	1	1.93	2.29	1.67
7	4	1	4	1	5.2	5.31	5.09
8	4	2	1	2	4.23	4.24	4.22
y_j1_*	6.16	8.98	8.01	14.5			
y_j2_*	6.97	6.61	5.39	13.3			
y_j3_*	5.23	6.66	6.81				
y_j4_*	9.43	5.54	7.58				
R*j*	4.2	3.44	2.62	1.25			
R*j*’	2.67	2.19	1.69	1.77			
Optimal level	A3	B4	C2	D2			
Primary and secondary order	A B D C						
Optimal parameter combination	A3 B4 D2C2						

Based on the range analysis method, the optimal combination obtained from the field orthogonal test was A3B4D2C2, which was consistent with the results obtained from the multiobjective orthogonal optimization test. The field test under the optimal combination A3B4D2C2 showed a cleaning performance with impurity and loss rates of 1.91% and 1.53%, respectively. This combination achieved the lowest impurity and loss rates among the orthogonal test combinations, thus achieving the goal of optimized cleaning performance.

### Comparison of the performance of cleaning screen before and after structural improvement

#### Comparison of airflow field evaluation indicators before and after optimization

To verify whether the values of the airflow field indicators had improved, measurements were taken under the optimal working parameter combination A3B4D2C2, which corresponds to a fan speed of 1250 r/min, first and second guide plate angles at the fan outlet of 37° and 30°, respectively, and a fish-scale screen opening of 24 mm. The airflow speed parallel to the sieve surface showed the same variation trend. Measurement test data obtained from the near-sieve plane were used to analyze the characteristics of the ideal airflow field distribution in the cleaning chamber of the cutting and longitudinal-flow combine harvester.

Using the above experimental plan and methods, we measured the airflow speed at the *Z* = 0 mm plane in the cleaning chamber under the working parameter combination used for normal rice harvesting. Table 12 presents the distribution of the airflow speed values along the X- and Y-axes in the cleaning chamber.

**Table 12:** Airflow velocity before improvement at each measuring point.

Y/mm	X/mm								
	50	150	250	350	450	550	650	750	850
300	0.32	0.35	0.29	0.34	0.31	0.33	0.42	0.34	0.35
450	1.76	3.78	2.86	2.43	2.25	2.58	3.51	4.21	3.12
600	4.78	5.43	5.21	4.82	4.35	4.65	5.38	6.02	5.29
750	3.35	4.98	4.87	3.65	2.35	3.21	4.76	5.26	4.25
900	2.48	3.94	3.57	2.76	1.87	2.54	3.21	4.31	3.05
1050	2.23	3.75	3.44	2.47	1.46	2.76	2.79	3.68	2.63
1200	3.12	4.75	4.15	3.51	2.43	3.54	3.87	4.33	3.26

Along the length of the sieve surface, the airflow speed in the cleaning chamber initially increased rapidly and then gradually decreased. At the rear end of the sieve, the airflow speed increased again. For the radial airflow distribution in the cleaning chamber, different *y* values along the sieve length were selected for the analysis. The airflow in the cleaning chamber showed a gradient distribution along the radial direction. As the measured plane moved further from the sieve surface, the airflow speed gradually decreased. This same variation trend was observed at different cross-sections of the entire cleaning chamber.

Based on the measured data, the evaluation values of the airflow field were calculated, resulting in a coefficient of variation of 0.202 for the lateral airflow uniformity, an average airflow speed of 2.79 m/s in the pre-sieve section, and an airflow increase of 1.71 m/s at the sieve tail.

Table 13 presents the distribution of the airflow speed values along the X- and Y-axes in the cleaning chamber after the improvement.

**Table 13:** Airflow velocity after improvement at each measuring point.

Y/mm	X/mm								
	50	150	250	350	450	550	650	750	850
300	2.16	3.87	3.52	3.12	2.31	3.22	3.64	4.01	2.37
450	5.28	6.25	5.67	5.61	4.36	5.62	5.78	6.43	5.46
600	6.05	6.32	6.21	5.87	5.01	5.94	6.32	6.57	5.78
750	4.21	4.75	4.56	4.38	4.21	4.44	4.67	4.87	4.31
900	3.68	4.56	4.32	4.21	3.56	4.11	4.45	4.75	3.97
1050	3.21	3.76	3.42	2.97	2.64	3.01	3.64	3.84	3.26
1200	4.16	4.67	4.32	3.89	3.76	4.11	4.46	4.87	4.21

After the structural improvement of the cleaning device, the airflow speed was significantly concentrated toward the front end of the sieve due to the streamlined arc plate structure. The distribution of the lateral airflow field along the sieve surface in the cleaning chamber could be characterized by higher airflow speeds near the walls and lower speeds in the middle. The maximum airflow speed in the pre-sieve section appeared approximately 150 mm from both the side walls, while the airflow speed at the center of the sieve was relatively low.

From the selected different X cross-sections, the longitudinal airflow speed distribution in the cleaning chamber showed that the airflow speed increased in the front section of the sieve. The maximum airflow speed occurred in the range of Y = 550–600 mm along the sieve length. In the central region of the cleaning chamber, between Y = 700 mm and 800 mm, a stable airflow speed region was observed. In the tail section of the sieve, the airflow speed increased again. Overall, the airflow speed along the length of the sieve surface first increased rapidly, then decayed and decreased. A narrow stable speed region existed at the center of the sieve, and the airflow speed increased again in the tail section.

Based on the measured data, the evaluation values of the airflow field were calculated, resulting in a coefficient of variation of 0.107 for the lateral airflow uniformity, an average airflow speed of 5.22 m/s in the pre-sieve section, and an airflow increase of 2.35 m/s at the sieve tail. These experimental results confirm the optimization of the airflow field.

#### Field performance testing of optimized cleaning screen

Table 14 presents the field test results under the optimal working parameter combination obtained by optimization.

As shown in Table 14, in the six sets of repeated tests conducted, the average seed loss rate before the structural improvement in the screening and cleaning processes was 2.46%, and the impurity rate of the seeds was 4.78%. After the structural improvement, the seed loss rate decreased significantly to only 0.78%, and the impurity rate reduced to 1.78%. The screening and cleaning performance had noticeably improved, and the results confirmed that the improvement made to the vibration screen structure helped achieve the optimization goal. This demonstrates the feasibility of improving the cleaning performance by enhancing the airflow in the airflow channel^54^.

**Table 14:** Field test results.

Test conditions test no.	Test field is 25 m long, harvesting speed is 1.2 m/s, and cutting width is 2 m							
	1		2	3	4	5	6	Average
Loss rate (%)	before	2.95	2.96	2.15	1.34	2.19	3.15	2.46
	after	0.61	1.14	0.51	1.22	0.4	0.82	0.78
Impurity rate (%)	before	4.3	5.2	5.6	4.3	5.7	3.6	4.78
	after	2.2	3	1	2.5	1.5	0.5	1.78

## Conclusions

1. A quantitative analysis into the airflow field in the cleaning chamber of a combine harvester was conducted by constructing three evaluation indicators: the average airflow speed in the pre-sieve section, the average increase in the airflow speed in the post-sieve section, and the uniformity of the lateral airflow. Combined with multiobjective orthogonal experiments and field trials, the validity of these indicators was verified, and the optimal parameter combination for the cleaning device was determined to be as follows: a fan speed of 1250 r/min, first and second guide plate angles of 37° and 30°, respectively, and a fish-scale sieve opening of 24 mm. This method can replace conventional field trials with airflow field measurements, significantly reducing the experimental cost and duration and providing a new approach for the parameter optimization of combine harvesters.
2. The application of streamlined arc plates to the shaking plate of the vibrating sieve and the design of a dynamic airflow guiding structure helped improve the airflow distribution characteristics within the cleaning chamber. After the improvement, the average airflow speed in the pre-sieve section increased to 5.22 m/s, the airflow increase in the post-sieve section reached 2.35 m/s, and the lateral airflow uniformity improved to 0.107. Field trials showed that the optimized structure significantly reduced the impurity rate (4.78%-1.78%) and loss rate (2.46%–0.78%), thus verifying the effectiveness of the airflow guiding design in enhancing the cleaning efficiency. This provides a practical basis for the structural improvement of transverse-flow combine harvesters.
3. The multiobjective optimization method for the airflow field balances parameters such as the fan speed, guide plate angle, and sieve opening through orthogonal experiments, achieving a comprehensive improvement in the cleaning performance. The results showed that the synergistic effect of airflow field optimization and structural improvement could significantly enhance the adaptability of combine harvesters, particularly in maintaining efficient cleaning even under high feed rates (3–6 kg/s). This method is not only applicable to rice but can also be extended to other grain harvesting scenarios, offering broad engineering application prospects.

## Data Availability

The data created and analyzed during the current study are available from the corresponding author upon reasonable request.
