# Correction: Optimization and experimental analysis of a cleaning device for super rice with high impurity rates based on airflow field enhancement

**DOI:** 10.1038/s41598-026-57235-5

**Published:** 2026-06-12

**Authors:** Guoqiang Wang, Fukai Wang, Yaquan Liang, Fang Li, Li Wang, Yuan Yuan

**Affiliations:** 1 Department of Agricultural Engineering, Jiangsu Agri-Animal Husbandry Vocational College, Taizhou, 225300 Jiangsu China; 2 State Key Laboratory of Intelligent Agricultural Power Equipment, Luoyang, 471039 Henan China; 3 Institute of Agricultural Facilities and Equipment, Jiangsu Academy of Agricultural Sciences, Nanjing, 210000 Jiangsu China; 4 Department of Mechanical Engineering and Mechanics, Xiangtan University, Xiangtan, 411105 Hunan China; 5 Department of Agricultural Engineering, Jiangsu University, Zhenjiang, 212013 Jiangsu China

Correction to: *Scientific Reports* 10.1038/s41598-026-40829-4, published online 29 March 2026

The original version of this Article contained errors in the Figures. Figure 6 was published as Figure 7 and Figure 7 was published as Figure 6. The Figure legends were correct at the time of publication.

The original Figures 6 and 7 and accompanying legends appear below.

**Fig. 6:**
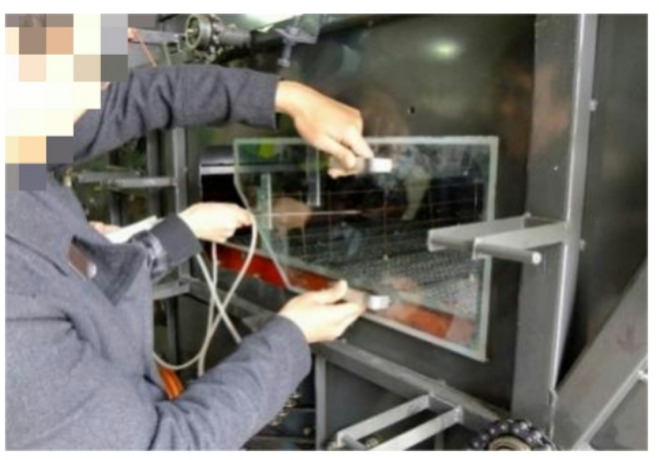
Distribution of measurement points in the cleaning chamber.

**Fig. 7:**
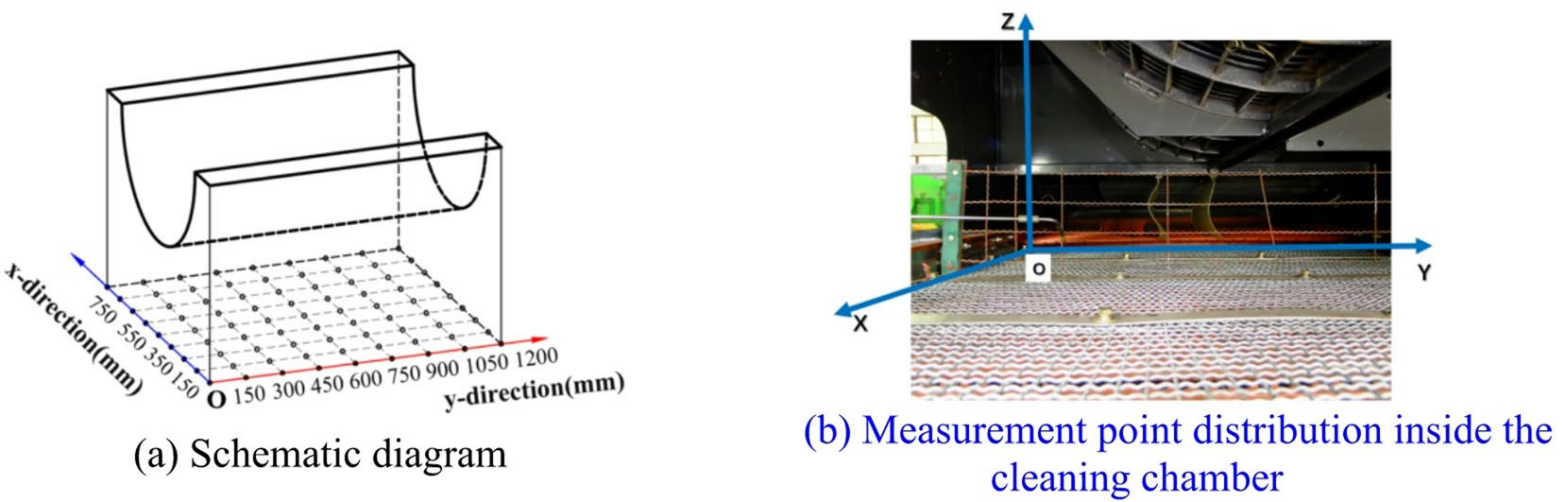
Indoor airflow field measurement inside the cleaning chamber.

Additionally, Yaquan Liang was incorrectly affiliated with Affiliation 2 and Fang Li was incorrectly affiliated with Affiliation 3.

Their correct affiliations are:

Yaquan Liang: 3. Institute of Agricultural Facilities and Equipment, Jiangsu Academy of Agricultural Sciences, Nanjing 210000, Jiangsu, China

Fang Li: 4. Department of Mechanical Engineering and Mechanics, Xiangtan University, Xiangtan 411105, Hunan, China.

The original Article has been corrected.

